# Effect of Voluntary Participation on Mobile Health Care in Diabetes Management: Randomized Controlled Open-Label Trial

**DOI:** 10.2196/19153

**Published:** 2020-09-18

**Authors:** Da Young Lee, Seung-Hyun Yoo, Kyong Pil Min, Cheol-Young Park

**Affiliations:** 1 Kangbuk Samsung Hospital Sungkyunkwan University School of Medicine Seoul Republic of Korea; 2 Korea University College of Medicine Seoul Republic of Korea; 3 National Health Insurance Service Wonju-Si Republic of Korea; 4 Huraypositive Inc Seoul Republic of Korea

**Keywords:** diabetes mellitus, health services research, mobile applications, diabetes, mHealth, app, lifestyle, self-management, volunteer, participation

## Abstract

**Background:**

The role of mobile health care (mHealth) in glycemic control has been investigated, but its impact on self-management skills and its psychological aspects have not been studied.

**Objective:**

We evaluated the efficacy of mHealth-based diabetes self-management education and the effect of voluntary participation on its effects.

**Methods:**

This study was a randomized controlled open-label trial conducted for 6 months at Kangbuk Samsung Hospital. Participants in the control group (n=31) maintained their previous diabetes management strategies. Participants in the intervention group (n=41) additionally received mHealth-based diabetes self-management education through a mobile app and regular individualized feedback from health care professionals. The primary outcome was change in glycated hemoglobin (HbA_1c_) level over 6 months between the 2 groups (intervention versus control) and within each group (at 6 months versus baseline). The secondary outcomes were changes in body mass index, blood pressure, lipid profile, and questionnaire scores (the Korean version of the Summary of Diabetes Self-Care Activities Questionnaire, an Audit of Diabetes Dependent Quality of Life, the Appraisal of Diabetes Scale, and Problem Areas in Diabetes) over 6 months between groups and within each group.

**Results:**

A total of 66 participants completed this study. HbA_1c_ (*P*=.04), total cholesterol level (*P*=.04), and Problem Areas in Diabetes scores (*P*=.02) significantly decreased; total diet (*P*=.03) and self-monitoring of blood glucose level scores (*P*=.01), based on the Summary of Diabetes Self-Care Activities Questionnaire, markedly increased within the intervention group. These significant changes were observed in self-motivated participants who were recruited voluntarily via advertisements.

**Conclusions:**

mHealth-based diabetes self-management education was effective at improving glycemic control and diabetes self-management skills and lowering diabetes-related distress in voluntary participants.

**Trial Registration:**

ClinicalTrials.gov NCT03468283; http://clinicaltrials.gov/ct2/show/NCT03468283

## Introduction

Lifestyle management, including diabetes self-management education, nutrition therapy, and physical activity, is a fundamental aspect of the successful management of diabetes [1,2]. Previous studies [1] have demonstrated that diabetes self-management education improves self-management, diabetes knowledge, satisfaction and quality of life, and glycemic control, and also reduces health care costs. However, only a small number of individuals eligible for diabetes self-management education receive it [3], and poor adherence has been reported [4]. Given that barriers to adherence might include patient factors, a patient-centered approach that would improve self-motivation could be useful.

Mobile health care (mHealth)–based diabetes self-management education has been regarded as an innovative option for diabetes self-management education, in that it may help to overcome time and location barriers and provide real-time individualized medical treatments [5-9]. The effectiveness of mHealth in diabetes management has been demonstrated in a few studies [6,10-13]. However, these previous studies [6,10-13] have mainly focused on the role of self-management education in glycemic control; the impact of mHealth on self-management skills and its psychological aspects have not been studied.

Therefore, we conducted this study to evaluate whether implementing mHealth-based diabetes self-management education could improve diabetes self-management and glycemic control and enhance patient quality of life. In addition, we aimed to investigate the importance of voluntary engagement in mHealth-based diabetes self-management education.

## Methods

### Study Participants

Patients with type 2 diabetes from an outpatient clinic at Kangbuk Samsung Hospital were invited to participate via advertisement in Kangbuk Samsung Hospital or via recommendation by their physician between June 2012 and July 2012 (Multimedia Appendix 1). Inclusion criteria were as follows: age ≥19 years; Android smartphone users; no changes in diabetes medication for at least 6 months; and HbA_1c_ levels ≥6.5% within the last 3 months. We recruited a total of 140 individuals (70 individuals for each group), such that the sample size afforded 80% power at a significance level of α=.05 with a 10% dropout rate, considering a mean difference in HbA_1c_ level of –0.50 based on prior research [5] investigating similar interventions. However, because many participants were excluded because they had an iPhone rather than an Android-based phone or had changed medications within 6 months, only 73 individuals were initially found to be eligible. Among these 73 individuals, 1 participant was excluded. Exclusion criteria were having a serious concomitant disease other than diabetes; a history of malignancy, myocardial infarction, cerebral infarction, or organ transplantation; being pregnant or planning for pregnancy within 6 months; planning to participate in other clinical studies; or illiteracy. Finally, 72 individuals were enrolled in this study.

All participants provided written informed consent before study procedures began. This study was reviewed and approved by the institutional review board of Kangbuk Samsung Hospital (KBS12089) and was carried out in accordance with the Helsinki Declaration. The trial was registered on February 26, 2018 with clinicaltrials.gov (NCT03468283). The CONSORT checklist [14] is available in Multimedia Appendix 2.

### Study Design and Details of the Intervention

This study was a randomized controlled open-label trial conducted from June 2012 to March 2013 at Kangbuk Samsung Hospital, Sungkyunkwan University, Seoul, Republic of Korea. Participants were randomly assigned to 1 of 2 groups: the intervention group or the control group. We used a computer-generated list of random numbers produced by a statistician with no clinical involvement in the trial to allocate the participants. Participants were allocated in the order in which they were registered.

Participants in the intervention group received mHealth-based diabetes self-management education, which consisted of the mobile app Healthynote (for Android; CVnet Co) and regular individualized feedback messages from health care professionals regarding their diabetes management. The intervention group uploaded the Healthynote app onto their own smartphone, were educated on how to use this service, and were provided a near field communication–enabled glucometer (CareSens N NFC; i-SENS Inc). They entered their medical information, such as self-monitored blood glucose level, dietary record, exercise, blood pressure, medication record, and body weight into the app and could check their data at any time by logging into the mobile app and could share it through social network services. The data entered in the app were automatically transmitted by wireless network to the server and stored on a secure website accessible only to providers. During the 6-month study period, participants in the intervention group received regular mobile messages from health care professionals, consisting of 2 endocrinologists and a nurse, via the mobile app once or twice a week. Messages contained general information about diabetes (eg, medications, diet, exercise, treatment goals), diabetes self-care behaviors based on recommendations from the American Diabetes Association [15] and the Korean Diabetes Association [16], encouragement, reminders if the patient had not used the app recently, and individualized advice based on their entered data. Participants could communicate bidirectionally with providers via app messages and call server administrators for technical problems. In the secure website for health care professionals, participant-generated health data were tracked and analyzed for trends and patterns. Using a function for sorting and filtering participants according to transmitted data, usage, and scheduled period, providers sent messages.

Participants in the control group maintained their previous diabetes management at Kangbuk Samsung Hospital. Providers were not involved with patient prescriptions. Among 7 endocrinologists at this center, 2 participated in this study as the principal investigator and co-investigator. Participants were recruited regardless of their endocrinologist. Providers telephoned participants in both groups to discuss research progress. Patients in the study did not have to make additional visits to the hospital beyond regular visits.

To evaluate the impact of self-motivation among our study participants, we divided the intervention group into 2 subgroups for analysis: a group of participants that voluntarily participated in the mobile diabetes self-management education program and joined after seeing an advertisement posted in the hospital (ie, the self-referred group, n=20) and a group who joined based on recommendation by their primary care physician (ie, the physician-referred group, n=19).

### Anthropometric and Laboratory Measurements

At baseline, all participants completed a self-administered questionnaire regarding demographic characteristics, social history, and other medical conditions. BMI was calculated as weight in kilograms divided by the square of height in meters. Blood pressure was measured twice using a standardized sphygmomanometer after 5 minutes of rest, and the lower values were used.

Venous blood samples were collected in the morning (between 8 AM and 9 AM) after an overnight fast of more than 8 hours. Concentrations of plasma glucose (fasting plasma glucose) were determined using the hexokinase method; glycated hemoglobin (HbA_1c_) level was determined using turbidimetric inhibition immunoassay; and lipid levels were determined using an enzymatic method and a homogeneous enzymatic calorimetric test. Biochemical values were measured using the Cobas Modular 6000 analyzer series (Roche Diagnostics). Methodology was aligned with Diabetes Control and Complications Trial and National Glycohemoglobin Standardization Program standards [17]. The coefficients of variation were 1.94% and 2.2% for HbA_1c_, 9.57% and 5.39% for cholesterol, and 7.54% and 4.14% for triglyceride level, for normal and abnormal values, respectively.

These variables were tested both at baseline and at 6 months. In addition, blood pressure, weight, lipid profile, fasting plasma glucose level, and HbA_1c_ level were also measured at 3 months.

### Questionnaires

To assess diabetes self-management, the impact of diabetes on quality of life, diabetes awareness, emotional stress derived from diabetes, and treatment satisfaction, participants answered 5 self-administered questionnaires both at baseline and at 6 months. The Korean version of the Summary of Diabetes Self-Care Activities Questionnaire (SDSCA) [18,19], the Audit of Diabetes Dependent Quality of Life (ADDQOL) [20,21], the Korean version of the Appraisal of Diabetes Scale (ADS) [22,23], the Problem Areas in Diabetes (PAID) questionnaire [24], and Diabetes Treatment Satisfaction Questionnaire status version (DTSQs) and change version (DTSQc) [25,26] were used.

### Statistical Analysis

We conducted a per protocol analysis. The primary outcomes were (1) difference (between the intervention and control groups) in HbA_1c_ levels at 6 months and (2) change (within each group) in HbA_1c_ levels from baseline at 6 months.

Two-tailed independent *t* tests, paired *t* tests, and chi-square tests were used to examine differences between the groups.

The comparisons of secondary outcomes—BMI, systolic blood pressure, diastolic blood pressure, lipid levels, and SDSCA, ADDQOL, ADS, and PAID scores between the 2 groups and within each group—were performed using Wilcoxon signed-rank tests and paired *t* tests. We compared the DTSQ scores of the 2 groups without analyzing longitudinal differences.

Additionally, we repeated the above-mentioned analysis after dividing the participants into control, voluntary, and physician-referred groups.

We calculated ΔHbA_1c_ by subtracting baseline HbA_1c_ level from the HbA_1c_ level measured at 6 months (lower ΔHbA_1c_ indicated better glycemic control). We conducted multivariate linear regression analysis adjusted for age, sex, baseline BMI, systolic blood pressure, HbA_1c_, high-density lipoprotein cholesterol level, and low-density lipoprotein cholesterol level to assess the relationship between ΔHbA_1c_ and the total frequency of self-monitoring of blood glucose level records, exercise records, diet records, medication records, and message reading rate for 6 months.

SPSS statistical software (version 22.0; IBM Corp) was used. A value of *P*<.05 was considered statistically significant. Bonferroni correction (for multiple comparisons) was used.

## Results

### Study Execution and Baseline Characteristics of Participants

**Figure 1:**
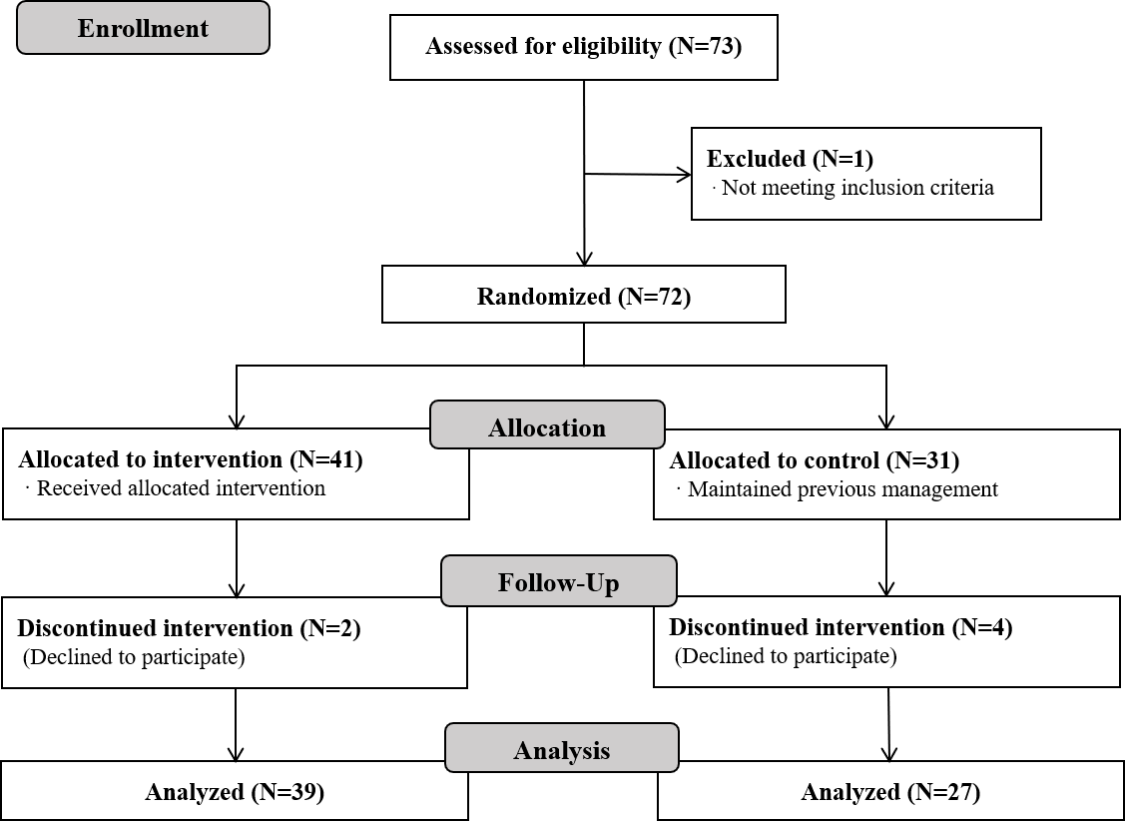
CONSORT flow diagram.

Among 72 participants, 66 completed the study (Figure 1); 2 participants from the intervention group and 4 participants from the control group were lost to follow-up.

There were no differences in metabolic parameters at baseline (Multimedia Appendix 3). Diabetes self-management status, especially diet and self-monitored blood glucose level, was superior in the control group.

### Changes in Metabolic Parameters and Questionnaire Scores

Although there was no significant difference between the control and intervention groups at 6 months (HbA_1c_: *P*=.05; total cholesterol *P*=.24), the intervention group showed statistical improvement in HbA_1c_ (*P*=.04) and total cholesterol levels (*P*=.04) after 6 months (Multimedia Appendix 4). SDSCA scores, especially total diet (*P*=.03) and self-monitoring of blood glucose level (*P*=.01), which were markedly increased compared with baseline, and DTSQ (change) scores at 6 months, which were significantly higher than those of the control group (*P*=.02). The PAID score significantly decreased during the study period in the intervention group, while there was no change in the control group.

As shown in Tables 1 and 2, the significant findings observed in the intervention group were concentrated in the self-referred group. Compared to baseline, HbA_1c_, total cholesterol levels, low-density lipoprotein cholesterol levels, total diet, and self-monitoring of blood glucose level on the SDSCA were only improved in the self-referred group. Compared to the physician-referred group, the self-referred group also had lower total cholesterol levels, triglyceride levels, exercise, and smoking scores on the SDSCA at 6 months.

**Table 1:** Changes in biochemical parameters over 6 months.

Parameter		Control (n=27)	Intervention				*P* value^a^		
			All (n=39)	Physician-referred (n=19)	Self-referred (n=20)	Physician-referred vs. control		Self-referred vs. control	Physician- vs. self-referred
**HbA_1c_^b^ (%)**									
	Baseline	7.5 (1.1)	7.4 (0.8)	7.3 (0.7)	7.4 (0.9)	.65		.92	.59
	3 months	7.3 (0.7)	7.1 (0.9)	7.0 (0.7)	7.2 (1.0)	.54		.94	.78
	6 months	7.6 (0.9)	7.1 (0.8)	7.2 (0.7)	7.1 (1.0)	.13		.09	.73
	*P* value^c,d^	.87	.04	.46	.04				
**BMI (kg/m^2^)**									
	Baseline	25.5 (3.0)	26.8 (4.2)	27.3 (4)	26.3 (3.7)	.24		.76	.44
	6 months	25.7 (3.1)	27.0 (4.1)	27.7 (4.7)	26.3 (3.4)	.09		.50	.31
	*P* value^c^	.08	.19	.06	.89				
**Systolic blood pressure (mmHg)**									
	Baseline	120.5 (12.0)	121.1 (14.1)	123.6 (13.5)	118.7 (14.5)	.71		.89	.28
	6 months	120.2 (11.0)	124.4 (12.0)	128.3 (11.6)	120.7 (11.4)	.02		.89	.05
	*P* value^c^	.91	.07	.10	.40				
**Total cholesterol (mg/dL)**									
	Baseline	150.2 (26.5)	148.7 (41.6)	156.2 (45.3)	141.5 (37.7)	.85		.70	.29
	6 months	149.5 (30.2)	139.8 (33.2)	153.4 (38.3)	128.4 (23.5)	.71		.01	.02
	*P* value^c^	.88	.04	.45	.04				
**Triglyceride level (mg/dL)**									
	Baseline	148.2 (67.5)	151.0 (113.5)	182.6 (143.6)	121.1 (65.9)	.46		.61	.11
	6 months	216.9 (206.6)	177.9 (116.8)	234.8 (146.3)	129.6 (49.9)	.76		.07	.01
	*P* value^c^	.10	.20	.18	.91				
**LDL C^e^ (mg/dL)**									
	Baseline	76.4 (18.0)	77.5 (25.6)	76.0 (23.1)	79.0 (28.3)	.99		.93	.73
	6 months	76.1 (18.2)	70.9 (20.7)	74.7 (24.1)	67.6 (17.2)	.83		.11	.30
	*P* value^c^	.91	.08	.77	.02				

^a^An independent *t* test was used.

^b^HbA_1c_: glycated hemoglobin.

^c^A paired *t* test or Wilcoxon signed-rank test was used.

^d^*P* value is for comparison between baseline and 6 months.

^e^LDL C: low-density lipoprotein cholesterol.

**Table 2:** Changes in the 5 questionnaires over 6 months.

Parameter			Control (n=27)		Intervention							*P* value^a^				
					All (n=39)		Physician-referred (n=19)		Self-referred (n=20)		Physician-referred vs. control		Self-referred vs. control		Physician- vs. self-referred	
**SDSCA^b^**																
	**Total diet**															
		Baseline	14.3 (4.4)		10.8 (3.9)		11.6 (3.0)		10.0 (4.4)		.11		<.001		.22	
		6 months	14.7 (5.2)		12.6 (5.3)		11.3 (3.3)		13.8 (6.5)		.01		.59		.15	
		*P* value^c^	.73		.03		.87		.02							
	**Exercise**															
		Baseline	7.2 (3.8)		6.0 (3.5)		5.4 (3.3)		6.6 (3.7)		.27		.84		.33	
		6 months	6.2 (4.4)		5.2 (3.7)		3.8 (2.7)		6.5 (4.1)		.03		.84		.02	
		*P* value^c^	.45		.16		.21		.44							
	**Self-monitoring of blood glucose**															
		Baseline	7.1 (4.7)		4.1 (4.9)		3.8 (5.4)		4.4 (4.5)		.10		.19		.42	
		6 months	6.7 (4.5)		6.6 (4.7)		5.1 (4.2)		8.0 (4.8)		.23		.36		.06	
		*P* value^c^	.35		.01		.24		.02							
	**Foot**															
		Baseline	4.1 (4.0)		3.8 (4.3)		3.2 (3.8)		4.3 (4.7)		.77		.98		.44	
		6 months	4.1 (4.0)		3.8 (4.3)		3.2 (3.8)		4.3 (4.7)		.46		.86		.43	
		*P* value^b^	>.999		.10		>.999		>.999							
	**Smoking**															
		Baseline	1.0 (2.2)		2.1 (3.2)		2.8 (3.5)		1.5 (2.9)		.10		.83		.20	
		6 months	1.7 (3.0)		2.1 (3.2)		3.2 (3.5)		1.1 (2.6)		.16		.41		.04	
		*P* value^c^	.10		.46		.18		.16							
**ADDQOL^d^**																
		Baseline	–3.3 (2.1)		–2.5 (1.5)		–2.7 (1.6)		–2.4 (1.6)		.54		.26		.59	
		6 months	–3.2 (1.7)		–2.8 (1.4)		–2.6 (1.4)		–2.9 (1.5)		.31		.59		.62	
		*P* value^c^	.58		.59		.67		.53							
**ADS^e^ total**																
		Baseline	15.5 (2.7)		15.8 (3.0)		15.7 (2.0)		15.9 (3.7)		.99		.91		.82	
		6 months	16.1 (2.4)		15.5 (3.2)		15.2 (2.7)		15.8 (3.6)		.24		.71		.58	
		*P* value^c^	.31		.48		.32		.86							
**PAID^f^**																
		Baseline	50.6 (9.8)		49.8 (15.2)		49.9 (13.5)		49.8 (16.9)		.98		.98		.99	
		6 months	47.5 (12.6)		44.2 (15.2)		42.4 (14.8)		45.9 (15.7)		.22		.70		.49	
		*P* value^c^	.30		.02		.09		.18							
**DTSQ^g^**																
		Baseline (DTSQs)	25.2 (4.7)		24.7 (6.1)		24.2 (6.5)		25.3 (5.8)		.80		.99		.59	
		6 months (DTSQc)	9.0 (4.6)		12.0 (4.7)		11.4 (4.6)		12.6 (4.9)		.10		.02		.47	

^a^An independent *t* test was used.

^b^SDSCA: Summary of Diabetes Self-Care Activities.

^c^A paired *t* test or Wilcoxon signed-rank test was used.

^d^ADDQOL: Audit of Diabetes Dependent Quality of Life.

^e^ADS: Appraisal of Diabetes Scale (Korean version).

^f^PAID: Problem Areas In Diabetes.

^g^DTSQc: Diabetes Treatment Satisfaction Questionnaire.

### Association Between Record Usage and ΔHbA_1c_

During the 6-month study period, a mean of 8.0 (SD 7.1) records per week of self-monitored blood glucose level, a mean of 0.9 (SD 1.6) records per week of exercise, and a mean of 1.5 (SD 1.6) records per week of diet were uploaded, and the message reading rate was a mean 54.0% (SD 33.4%). Participants in the self-referred group used the mobile app more than those in the physician-referred group (Table 3). In multivariate linear regression analysis, the correlations of self-monitored blood glucose coefficient, exercise record, diet record, and message reading rate with ΔHbA_1c_ were –0.002 (*P*<.001), –0.008 (*P*=.08), –0.005 (*P*=.07), and –0.005 (*P*=.10), respectively.

**Table 3:** Mobile app usage and correlation with ΔHbA_1c_ in the intervention group.

Factors	Physician-referred (n=19)	Self-referred (n=20)	*P* value^a^	Standardized coefficient (β)	*P* value^b^
Self-monitoring of blood glucose level record (per week)	5.3 (5.0)	10.6 (8.0)	.02	–0.442	.01
Exercise record (per week)	0.4 (0.9)	1.4 (1.9)	.04	–0.308	.08
Diet record (per week)	1.3 (1.7)	1.7 (1.5)	.43	–0.312	.07
Message reading rate	46.9 (33.2)	61.1 (33.0)	.19	–0.273	.10

^a^An independent *t* test was used.

^b^Linear regression with adjustments for age, sex, and baseline BMI, systolic blood pressure, HbA_1c_ level, high-density lipoprotein cholesterol level, and low-density lipoprotein cholesterol level.

### Adverse Events

There were 4 adverse events (2 in the intervention group and 2 in the control group) during the study period: an episode of depression, hospital admission due to aggravation of glycemic control, herpes zoster, and a traffic accident. However, these 4 patients completed the study.

## Discussion

This prospective study showed that mHealth-based diabetes self-management education helps to improve glycemic status and diabetes self-management skills, reduces diabetes-related distress, and provides higher user satisfaction, especially in voluntary participants.

Although over 1100 diabetes-related smartphone apps are currently available [27], and prior studies [6,10-13,28,29] have emphasized their impact on HbA_1c_, there are limited data available on improving self-care skills or other clinical endpoints in patients with type 2 diabetes [30].

In other chronic diseases such as heart failure, chronic lung disease, or cardiovascular disease, mHealth technologies have demonstrated potential for facilitating adherence to chronic disease management [31], as well as improving lipid levels and weight control [32,33].

While mHealth-based diabetes self-management education provides helpful reminders and behavioral reinforcements for adherence to complex care regimens such as medications, blood glucose level checking, and secondary prevention testing [5], weekly health counseling via app messages provides regular contact with health professionals and could have strengthened self-management and behavior changes [34], in addition to offering emotional support and detailed reflection. The characteristics of this study were in line with the 4 key elements of technology-based diabetes management interventions for HbA_1c_ decrement that were suggested by Greenwood et al [35]: (1) 2-way communication, (2) analysis of patient-generated health data, (3) tailored education, and (4) individualized feedback.

Most clinically meaningful changes observed in the intervention group were seen in the voluntary (active engagement) group (Tables 1 and 2). The proportion of primary care physicians who participated in this study as investigators in the self-referred group (2/20, 10%) was significantly lower (*P*<.001 from chi-square analysis) than that of the physician-referred group (13/19, 68.4%). This reflects a significantly higher level of self-motivation for diabetes management among participants within the self-referred group. These findings are in line with previous evidence that initial active engagement in self-monitoring with a telemonitoring device contributed to excellent glycemic improvement [36].

After a slight decrement in HbA_1c_ levels over the first 3 months (Table 1), this improvement was maintained only in the self-referred group at 6 months, resulting in a significant decrement versus baseline (*P*=.04); this phenomenon is referred to as the Hawthorne effect [37]. Significance of the self-referred group (*P*=.04) was related to the impact of mHealth-based diabetes self-management education. This characteristic is important to understanding who uses apps and what keeps them engaged [38] and can be used to select participants for large-population mHealth services in the future. Advanced model cycling in intensive mHealth-based diabetes self-management education and a self-application period after selecting highly active individuals will be commercially applicable to managing more participants in the future. The degree of improvement in HbA_1c_ was less than that seen for other mHealth models for diabetes [5,6,10-13]. However, as a higher baseline HbA_1c_ level is correlated with a greater decrease in HbA_1c_ in response to various antidiabetes management methods [39], a slight improvement in HbA_1c_ level was predicted in this study, which had a mean baseline HbA_1c_ level of 7.4% (SD 0.8%). In prior studies with a baseline HbA_1c_ level <8.0%, the decrement in HbA_1c_ was approximately 0.4% [6,10].

Frequent app use, especially frequent self-monitored blood glucose level, showed a notable positive impact in our study, as reported in previous studies [40]. Through the ease and accuracy of blood glucose level tracking for patients via a near field communication–enabled glucometer, participants were encouraged to perform more self-monitored blood glucose level. There was also a possible correlation between the number of exercise records and diet records with ΔHba_1c_, although nonsignificance was indicated (*P*=.08 and *P*=.07, respectively; Table 3).

Despite the previously mentioned strengths of this study, several limitations should be considered. First, the number of participants was small, and the researchers were not blinded, given that this study was an open-label study. Second, unlike the selection of the intervention group, the stratification of physician-referred and self-referred groups could not be randomized. The physician-referred and self-referred groups might differ in terms of their familiarity with the use of digital equipment, as participant digital proficiency and comprehension of the mHealth-based diabetes self-management education were not considered at baseline or during the follow-up period. Third, we conducted per-protocol analysis, rather than intention-to-treat analysis. There were no data available after the baseline exam for the 2 participants who declined to participate in intervention group. Because we thought that mHealth-based diabetes self-management education was applied in those participants, we decided to exclude them from analysis. Fourth, this trial was registered at clinicaltrials.gov after the end of the study. Finally, although the real comparator between unmotivated and motivated groups would be among people who declined to participate in this study, we have no way of obtaining information about those patients.

In conclusion, this prospective study demonstrated that an mHealth-based diabetes self-management education that supports diabetes self-management through health counseling messages leads to improved glycemic status and diabetes self-management skills, reduced diabetes-related distress, and high user satisfaction. These positive impacts were predominantly observed in participants who engaged in this mobile app in a voluntary manner and in those who utilized the app more frequently.

## Appendix

Multimedia Appendix 1 Study design.

Multimedia Appendix 2 CONSORT checklist.

Multimedia Appendix 3 Baseline characteristics and comparisons between the three groups.

Multimedia Appendix 4 Changes in biochemical parameters and six questionnaires in two groups over six months.

## Abbreviations

ADDQOL: Audit of Diabetes Dependent Quality of Life
ADS: Appraisal of Diabetes Scale
DTSQ: Diabetes Treatment Satisfaction Questionnaire
HbA_1c_: glycated hemoglobin
mHealth: mobile health care
PAID: Problem Areas in Diabetes
SDSCA: Summary of Diabetes Self-Care Activities
