# Supplementary figures and images for: Effect of Voluntary Participation on Mobile Health Care in Diabetes Management: Randomized Controlled Open-Label Trial
Source: JMIR Mhealth Uhealth. 2020 Sep 18;8(9):e19153. doi: 10.2196/19153 (PMC7532462; doi:10.2196/19153)

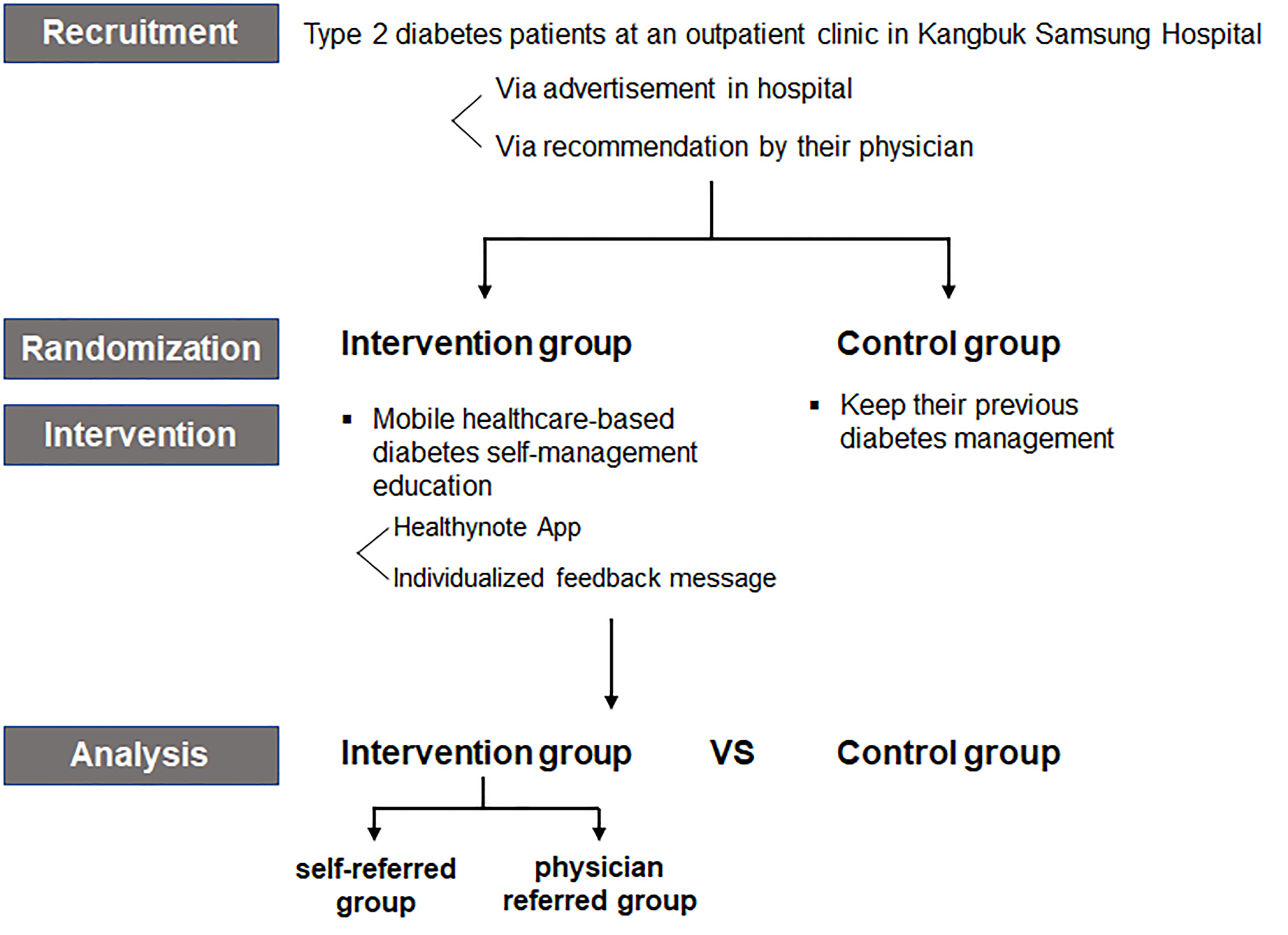

Supplement: Multimedia Appendix 1 [file mhealth_v8i9e19153_app1.png]
