# Supplementary material for: Effect of Voluntary Participation on Mobile Health Care in Diabetes Management: Randomized Controlled Open-Label Trial
Source: JMIR Mhealth Uhealth. 2020 Sep 18;8(9):e19153. doi: 10.2196/19153 (PMC7532462; doi:10.2196/19153)
Supplement: Multimedia Appendix 3 [file mhealth_v8i9e19153_app3.docx]

**Multimedia Appendix 3.** Baseline characteristics and comparisons between the three groups

|  | | Control (*n*=27) | Intervention | | | | | *P*^b^ from ANOVA |
| --- | --- | --- | --- | --- | --- | --- | --- | --- |
|  |  |  | Total  (*n*=39) | *P^a^* vs. control | Physician referred  (*n*=19) | Self-referred  (*n*=20) | *P*^a^ (Physician vs. Self-referred) |  |
| Age | | 53.2±8.2 | 50.0±7.9 | .12 | 47.7±6.3 | 52.1±8.9 | .09 | .30 |
| Sex, male (%) | | 16 (59.3) | 32 (82.1) | .05 | 17 (89.5) | 15 (75.0) | .24 | .07 |
| DM duration | | 9.5±6.7 | 10.0±6.2 | .78 | 8.5±6.7 | 11.4±5.5 | .16 | .94 |
| BMI (kg/m²) | | 25.5±3.0 | 26.8±4.2 | .15 | 27.3±4.7 | 26.3±3.7 | .44 | .23 |
| SBP (mmHg) | | 120.5±12.0 | 121.1±14.1 | .85 | 123.6±13.5 | 118.7±14.5 | .28 | .77 |
| HbA1c (%) | | 7.5±1.1 | 7.4±0.8 | .48 | 7.3±0.7 | 7.4±0.9 | .59 | .30 |
| TC (mg/dL) | | 150.2±26.5 | 148.7±41.6 | .86 | 156.2±45.3 | 141.5±37.7 | .29 | .85 |
| TG (mg/dL) | | 148.2±67.5 | 151.0±113.5 | .90 | 182.6±143.6 | 121.1±65.9 | .11 | .97 |
| LDL-C (mg/dL) | | 76.4±18.0 | 77.5±25.6 | .85 | 76.0±23.1 | 79.0±28.3 | .73 | .61 |
| High education^§^ | | 24 (88.9) | 37 (94.9) | .49 | 17 (89.5) | 20 (100) | .23 | .31 |
| Full-time employee | | 12 (44.4) | 33 (84.6) | .01 | 16 (84.2)^c^ | 17 (85.0)^c^ | .64 | .01 |
| SDSCA | |  |  |  |  |  |  |  |
|  | Total diet | 14.3±4.4 | 10.8±3.9 | .01 | 11.6±3.0^c^ | 1.0±4.4^c^ | .22 | .01 |
|  | Exercise | 7.1±3.8 | 6.0±3.5 | .22 | 5.4±3.3 | 6.6±3.7 | .33 | .30 |
|  | SMBG | 7.0±4.7 | 4.1±4.9 | .03 | 3.8±5.4 | 4.4±4.5 | .42 | .08 |
|  | Foot | 4.1±4.0 | 3.8±4.3 | .79 | 3.2±3.8 | 4.3±4.7 | .44 | .62 |
|  | Smoking | 1.0±2.2 | 2.1±3.2 | .10 | 2.8±3.5 | 1.5±2.9 | .20 | .16 |
| ADDQOL | | -3.3±2.1 | -2.5±1.5 | .12 | -2.7±1.6 | -2.4±1.6 | .59 | .26 |
| ADS total | | 15.5±2.7 | 15.8±3.0 | .73 | 15.7±2.0 | 15.9±3.7 | .82 | .85 |
| PAID | | 50.6±9.8 | 49.8±15.2 | .82 | 49.9±13.5 | 49.8±16.9 | .99 | .96 |
| DTSQs | | 25.2±4.7 | 24.7±6.1 | .72 | 24.2±6.5 | 25.3±5.8 | .59 | .87 |

Data are presented as mean ± SD or frequency (%).

ADDQOL, audit of diabetes dependent quality of life; ADS, the Korean version of the appraisal of diabetes scale; BMI, body mass index; DTSQs, the status version of the diabetes treatment satisfaction questionnaire; HbA1c, glycated hemoglobin; LDL-C, low density lipoprotein cholesterol; PAID, the problem areas in diabetes; SBP, systolic blood pressure; SDSCA, self-care activities questionnaire; TC, total cholesterol; TG, triglyceride.

^a^*P* value by Student’s *t*-test. ^b^*P* values were derived from ANOVA. ^c^*P* value<0.05 vs. control. ^§^High school and above.
