# Supplementary material for: Effect of Voluntary Participation on Mobile Health Care in Diabetes Management: Randomized Controlled Open-Label Trial
Source: JMIR Mhealth Uhealth. 2020 Sep 18;8(9):e19153. doi: 10.2196/19153 (PMC7532462; doi:10.2196/19153)
Supplement: Multimedia Appendix 4 [file mhealth_v8i9e19153_app4.docx]

**Multimedia Appendix 4.** Changes in biochemical parameters and six questionnaires in two groups over six months

| Variable | | Control Group (*n*=27) | | | Intervention Group (*n*=39) | | | *P*^b^ |
| --- | --- | --- | --- | --- | --- | --- | --- | --- |
|  |  | Baseline | 6 months | *P*^a^ | Baseline | 6 months | *P*^a^ |  |
| BMI (kg/m²) | | 25.5±3.0 | 25.7±3.1 | .08 | 26.8±4.2 | 27.0±4.1 | .19 | .17 |
| SBP (mmHg) | | 120.5±12.0 | 120.2±11.0 | .91 | 121.1±14.1 | 124.4±12.0 | .07 | .15 |
| HbA1c (%) | | 7.5±1.1 | 7.6±0.9 | .87 | 7.4±0.8 | 7.1±0.8 | .04 | .05 |
| TC (mg/dL) | | 150.2±26.5 | 149.5±30.2 | .88 | 148.7±41.6 | 139.8±33.2 | .04 | .24 |
| TG (mg/dL) | | 148.2±67.5 | 216.9±206.6 | .10 | 151.0±113.5 | 177.9±116.8 | .20 | .34 |
| LDL-C (mg/dL) | | 76.4±18.0 | 76.1±18.2 | .91 | 77.5±25.6 | 70.9±20.7 | .08 | .30 |
| SDSCA | |  |  |  |  |  |  |  |
|  | Total diet | 14.3±4.4 | 14.7±5.2 | 0.73 | 10.8±3.9 | 12.6±5.3 | .03 | .13 |
|  | Exercise | 7.2±3.8 | 6.2±4.4 | 0.45 | 6.0±3.5 | 5.2±3.7 | .16 | .36 |
|  | SMBG | 7.1±4.7 | 6.7±4.5 | 0.35 | 4.1±4.9 | 6.6±4.7 | .01 | .95 |
|  | Foot | 4.1±4.0 | 4.1±4.0 | 0.10 | 3.8±4.3 | 3.8±4.3 | .10 | .79 |
|  | Smoking | 1.0±2.2 | 1.7±3.0 | 0.10 | 2.1±3.2 | 2.1±3.2 | .46 | .69 |
| ADDQOL | | -3.3±2.1 | -3.2±1.7 | .58 | -2.5±1.5 | -2.8±1.4 | .59 | .35 |
| ADS total | | 15.5±2.7 | 16.1±2.4 | .31 | 15.8±3.0 | 15.5±3.2 | .48 | .41 |
| PAID | | 50.6±9.8 | 47.5±12.6 | .30 | 49.8±15.2 | 44.2±15.2 | .02 | .36 |
| DTSQs & DTSQc | | 25.2±4.7 | 9.0±4.6 |  | 24.7±6.1 | 12.0±4.7 |  | .02 |

Data are presented as mean±SD or frequency (%).

ADDQOL, audit of diabetes dependent quality of life; ADS, the Korean version of the appraisal of diabetes scale; BMI, body mass index; DTSQc, the change version the diabetes treatment satisfaction questionnaire; DTSQs, the status version of the diabetes treatment satisfaction questionnaire; HbA1c, glycated hemoglobin; LDL-C, low density lipoprotein cholesterol; PAID, the problem areas in diabetes; SBP, systolic blood pressure; SDSCA, self-care activities questionnaire; TC, total cholesterol; TG, triglyceride.

^a^*P* value vs. baseline by paired *t*-test or Wilcoxon signed rank test within each group. ^b^*P* value (control vs. intervention) by Student’s *t*-test at 6 months.
